# Ameliorative effect of nebivolol in doxorubicin-induced cardiotoxicity

**DOI:** 10.25122/jml-2023-0090

**Published:** 2023-09

**Authors:** Hussein Al-Amir, Ali Janabi, Najah Rayish Hadi

**Affiliations:** 1 Directorate of Najaf Health, Najaf, Iraq; 2 Department of Pharmacology and Toxicology, Faculty of Pharmacy, University of Kufa, Najaf, Iraq; 3 Department of Pharmacology and Therapeutics, Faculty of Pharmacy, University of Kufa, Najaf, Iraq

**Keywords:** doxorubicin, cardiotoxicity, nebivolol, inflammatory mediators, oxidative stress, apoptotic factors

## Abstract

This study aimed to investigate the potential of nebivolol in preventing doxorubicin-induced cardiotoxicity by targeting the inflammatory, oxidative, and apoptotic pathways. Twenty-eight male rats were randomly divided into four groups, each consisting of seven rats. The control group received standard diets and unrestricted access to water. The rats in the normal saline (N/S) group were administered a 0.9% normal saline solution for two weeks. The doxorubicin group (the “induced group”) received doxorubicin at a dosage of 2.5 mg/kg three times per week for two weeks. The nebivolol group received an oral dose of 4 mg/kg of nebivolol for the same duration. The cardiac tissues of rats treated with doxorubicin exhibited increased levels of tumor necrosis factor, interleukin-1, malondialdehyde, and caspase-3 compared to the normal saline control group (p<0.05), along with decreased levels of total antioxidant capacity and Bcl-2. These results show that doxorubicin is harmful to the heart. The administration of nebivolol significantly reduced the cardiotoxic effects induced by doxorubicin, as indicated by a statistically significant decrease in the levels of inflammatory markers, specifically tumor necrosis factor-alpha (TNF-α) and interleukin-1 beta (IL-1β) (p<0.05). The nebivolol group exhibited a significant decrease in malondialdehyde levels, which serves as a signal of oxidation, in cardiac tissue compared to the doxorubicin-only group (p<0.05). Additionally, the nebivolol group showed a significant increase in overall antioxidant capacity. Nebivolol dramatically attenuated doxorubicin-induced cardiotoxicity in rats, likely by interfering with oxidative stress, the inflammatory response, and the apoptotic pathway.

## INTRODUCTION

Cardiotoxicity is one of the most serious adverse effects of various chemotherapy medications, and it greatly increases morbidity and mortality rates [1]. The manifestations of cardiotoxicity are diverse, encompassing subtle alterations in blood pressure, the development of arrhythmias, and the onset of cardiomyopathy. The indications and symptoms of myocardial dysfunction span a spectrum, ranging from mild to severe, potentially culminating in enduring cardiac insufficiency or fatality [2]. Chemotherapy-induced cardiotoxicity has several pathways, including biological processes. Both the generation of free oxygen radicals and the stimulation of immunogenic responses cause harm. The heart contains antigen-presenting cells (APCs) [3, 4]. Doxorubicin (Dox) is an anthracycline generated by the *Streptomyces peucetius var caesius* mutant strain. It is a well-known and widely used antineoplastic medicine in treating various diseases, including breast cancer, leukemia, and pediatric cancer [5]. Doxorubicin works as an anti-cancer agent in a number of ways, including DNA intercalation and topoisomerase II inhibition, which can result in cell death or cell growth arrest [6, 7]. Doxorubicin discovery in the 1960s was a watershed moment in cancer research. When it was first introduced in the 1970s, it was intended to treat a variety of human malignancies, including cancer. Since then, it has emerged as one of the most often-used drugs in this category. The main adverse effect that limits the use of this medication is cardiomyopathy, which can result in congestive heart failure and death [8]. The mechanism of anthracycline-induced cardiomyopathy is currently being disputed and is unknown. Various molecular pathways have been proposed to explain anthracycline cardiotoxicity over the years, but no single explanation appears to contain all of the existing fundamental and clinical facts [9]. Numerous factors, such as oxidative stress, inflammation, mitochondrial damage, endoplasmic reticulum (ER) stress, calcium (Ca^2+^) imbalance, apoptosis, fibrosis, and autophagy dysregulation, have been linked to DOX-induced cardiotoxicity [10, 11]. Nebivolol is a beta-adrenoceptor antagonist of the third generation that specifically blocks 1 -ARs and produces vasodilation via NO. It was first developed, patented, and approved for medical use in Europe in 1997 but gained FDA approval for hypertension treatment in the United States in 2007 [12]. Compared to other beta-adrenoceptor antagonists, nebivolol has the highest selectivity for 1-receptors [13]. The mechanism of action of the racemate nebivolol involves D-nebivolol inhibiting 1-ARs in the heart's conducting tissue and myocyte. Several investigations have shown that L-nebivolol causes an increase in nitric oxide (NO) availability. However, the precise process is unknown [14]. All of these variables lead to increased endothelial nitric oxide synthase (eNOS) activity and thus greater nitric oxide (NO) emission. Nebivolol has also been shown to enhance thermogenic and mitochondrial genes via 3-AR, activate lipolysis, and activate lipolysis [15]. Although the 3-ARs have not yet been thoroughly investigated, preliminary data suggests that they can activate a number of signaling pathways related to heart protection. Hence, concentrating on 3-ARs may be a novel strategy to improve metabolism and cardiac function [16]. Adrenergic blockers may provide only minimal protection from the cardiotoxicity of doxorubicin [17]. In particular, third-generation -blockers are intriguing. Nebivolol interacts with the L-arginine/nitric oxide pathway, which gives rise to its antioxidant and vasodilatory properties [18].

This study aimed to examine the potential protective effects of nebivolol in mitigating doxorubicin-induced cardiotoxicity by inhibiting pro-inflammatory, oxidative, and apoptotic pathways.

## Material and Methods

### Animal preparation

The study utilized a sample of 28 male Sprague Dawley rats obtained from the Faculty of Science, University of Kufa. The rats had an average weight ranging from 150 to 240 grams and were at a maturation stage of 10 to 12 weeks. The Kufa College of Science animal facility served as the designated location for housing laboratory rats. The subjects were housed in an isolated group enclosure equipped with environmental controls to maintain a consistent humidity and temperature of 24±2 C°. Water and a standard diet were provided to the animals' enclosures based on their feed intake. In order to facilitate the recovery of animals from the physiological and psychological strain induced by the alteration in their habitat, a pharmacological intervention was administered to them, followed by a further period of isolation lasting for two weeks.

### Study design

A total of 28 male rats, aged three months, with weights ranging from 150 to 240 grams, were selected for this study. The rats were randomly assigned to four groups, with each group consisting of seven rats. The control group of rats was provided with a diet of natural ingredients and water ad libitum throughout the study. The rats in the normal saline (N/S) group were administered an oral dose of 10 ml/kg/day of 0.9% normal saline for two weeks. Nebivolol was administered with a regular saline solution. The doxorubicin group (cardiotoxicity induction) received intraperitoneal (IP) administration of doxorubicin at a dosage of 2.5 mg/kg three times a week for two weeks, resulting in a cumulative dose of 15 mg/kg. [19]. The administration of nebivolol was conducted orally within the nebivolol group, also referred to as the treatment group, at a dosage of 4 mg/kg/day for two weeks [20].

### Collection of samples

#### Blood sample collection

The body weight of each animal was documented 48 hours after administering the previous dose of anticancer treatment. For anesthesia induction, the animals received an intravenous injection of 100 mg/kg of ketamine and 10 mg/kg of xylazine. Following the administration of general anesthesia, the surgical procedure involved an incision at the designated site. A thoracoabdominal incision was then carefully performed, providing direct access to the left ventricle of the heart through a small opening. Blood samples were collected from this location and placed in clot activator gel tubes. The serum was successfully isolated following a 10-minute centrifugation at a rotational speed of 4,000 revolutions per minute. Subsequently, the enzyme-linked immunosorbent assay (ELISA) test was performed using commercially available testing kits for Interleukin-1β (IL-1β) and tumor necrosis factor α (TNF-α), with the obtained serum serving as the test sample.

#### Tissue sample preparation

The basal aspect of the heart was rinsed with saline solution at a low temperature to eliminate any erythrocytes or thrombi. It was subsequently preserved at a significantly low temperature (-80 °C), weighed following thawing, and subjected to homogenization using an ultrasonic liquid processor at a high level of intensity. The homogenization was performed in a 1:10 (weight/volume) solution of 0.1 M phosphate buffer saline (pH 7.4), supplemented with 1% Triton-100 and a protease [21]. To quantify the levels of caspase-3, malondialdehyde (MDA), total antioxidant capacity (TAC), and B-cell lymphoma 2 (Bcl2), commercially available ELISA kits were utilized. The supernatants were collected and subjected to homogenization, followed by centrifugation at 14000 rpm for 10 minutes at a temperature of 4 degrees Celsius, per the manufacturer's instructions (PARS Biochem, China).

#### Histopathology tissue sampling

The highest region was conserved and treated with a 10% neutral formalin solution, enclosed within a paraffin block, and sliced into 5 micrometers thick sections for histological examinations with a microtome. Light microscopy examined tissue sections subjected to hematoxylin and eosin staining [22].

### Quantification of key parameters

IL-1β, TNF-α, casp-3, Bcl2, MDA, and TAC concentrations were quantified using enzyme-linked immunosorbent assay (ELISA) kits per the manufacturer's instructions.

### Statistical analysis

The data analysis was performed using GraphPad Prism 8 software. The data is presented as the mean and standard error of the mean (SEM). A one-way analysis of variance (ANOVA) was employed to conduct a comparison among all the groups. Histopathological alterations in distinct groups were assessed using post-hoc testing with the Bonferroni multiple comparison test following one-way ANOVA. A significance level of p<0.05 was determined for all tests, indicating statistical significance.

## RESULTS

**Figure 1:**
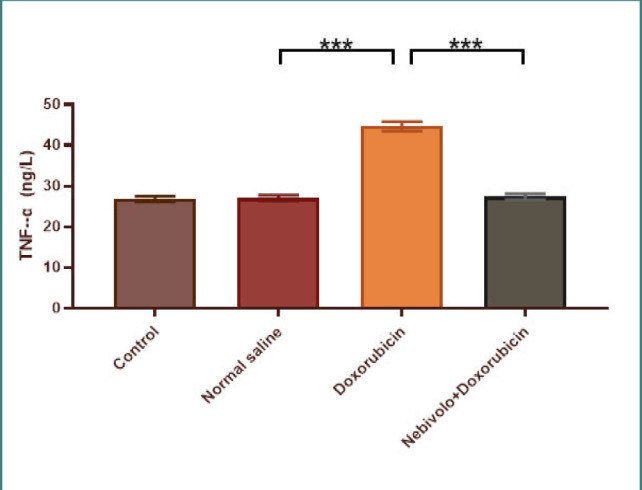
Serum TNF-α levels in experimental groups

**Figure 2:**
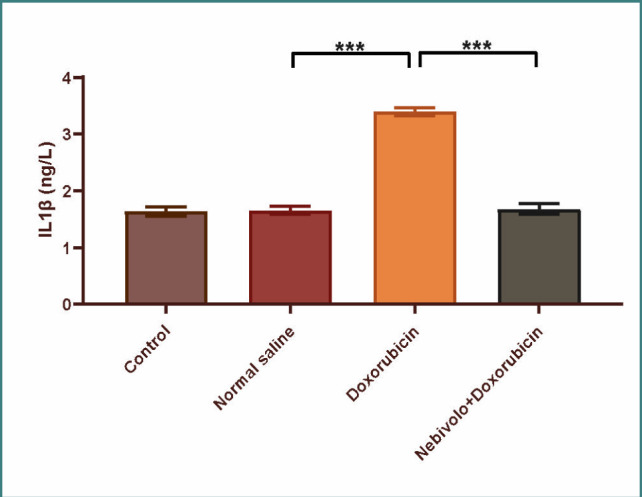
Serum IL-1β levels in experimental groups

**Figure 3:**
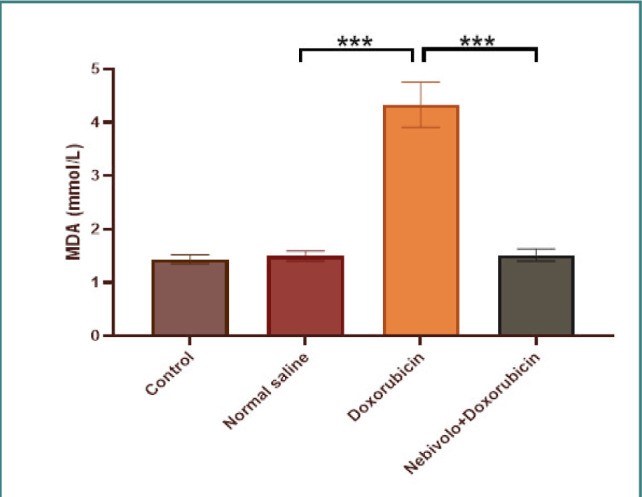
Cardiac MDA levels in experimental groups

**Figure 4:**
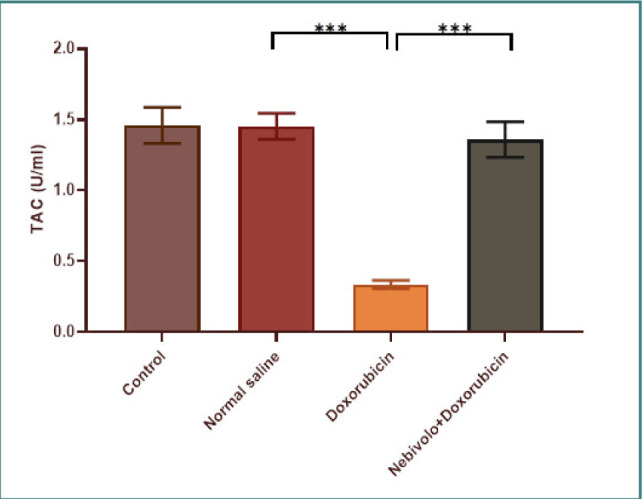
Experimental group cardiac TAC

**Figure 5:**
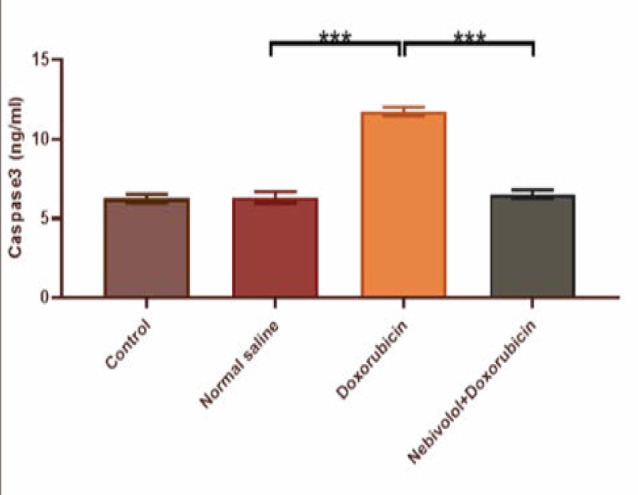
Caspase 3 levels in the experimental groups

**Figure 6:**
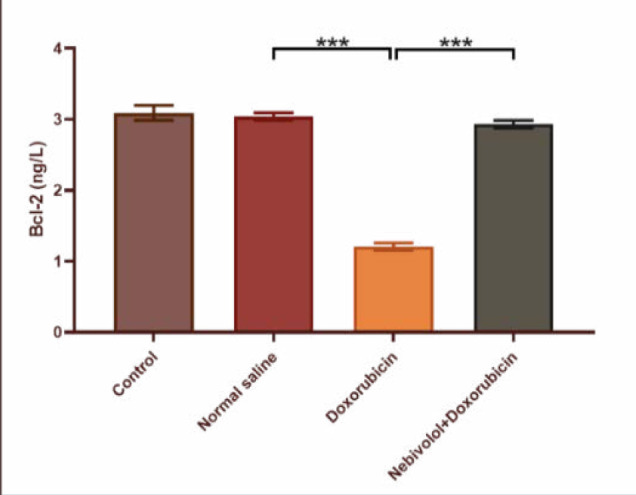
Bcl-2 levels in the expearimental groups

**Figure 7:**
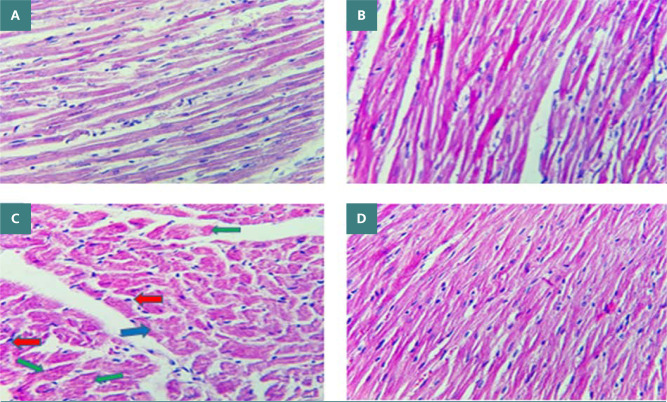
A: The myocardium of the control group exhibits normal histology, indicating the absence of any injury. B: The myocardium of the vehicle group, which was treated with normal saline, exhibited an absence of damage, indicating a normal histological appearance. C: Administration of doxorubicin resulted in significant histological impairment to the rat's heart. This was demonstrated by the presence of disorganized myocardial fibrils, stromal edema, necrosis, pyknotic nuclei (indicated by a red arrow), perinuclear halo (indicated by a blue arrow), and fading of nuclei (indicated by a green arrow). D: The myocardium in rats treated with doxorubicin (2.5 mg/kg) and nebivolol (4 mg/kg) reveals normal histology, well-organized myocardial fibrils, and appropriate staining. H&E staining was employed for histological assessment.

Cardiotoxicity was observed due to the administration of doxorubicin at a dosage of 2.5 mg/kg. The doxorubicin group exhibited a large increase in TNF-α, IL-1β, MDA, and caspase-3 levels, while the N/S group's cardiac tissues showed a significant decrease in TAC and Bcl-2 levels. The data presented in Figures 1 and 2 demonstrate that nebivolol effectively mitigated doxorubicin-induced cardiotoxicity, as evidenced by the decreased levels of the inflammatory mediators TNF-α and IL-1β. The levels of the oxidative marker MDA were significantly lower in cardiac tissue (Figure 3) and significantly higher in TAC (Figure 4) in the group treated with nebivolol compared to the group treated with doxorubicin alone. In addition, the administration of nebivolol resulted in a significant decrease in doxorubicin-induced apoptosis, as evidenced by the observed reduction in cardiac caspase-3 levels (Figure 5) and a notable elevation in Bcl-2 expression compared to the group treated with doxorubicin alone (Figure 6). In addition, nebivolol exhibited a significant improvement in the scores of histological lesions associated with cardiomyopathy, as compared to the group treated with doxorubicin (Table 1 and Figure 7 A-D).

**Table 1:** Histopathological scores

Group	SEM±Mean	Comparison	p-value
Control	0±0	Normal saline vs. control	>0.9999
Normal saline	0±0	Normal Saline vs. Nebivolol	0.5747
Doxorubicin	3.76±0.18	Doxorubicin vs. Normal saline	<0.0001
Nebivolol	0.85±0.26	Doxorubicin vs. Nebivolol	0.0776

The groups included rats given a vehicle (normal saline), doxorubicin, doxorubicin with nebivolol, or no treatment. The data is displayed as mean values with standard error (SEM). Statistical analysis revealed a highly significant difference (p<0.001) among the groups using one-way ANOVA with Bonferroni multiple comparison test.

Serum IL-1β levels were compared among the vehicle, doxorubicin, doxorubicin + nebivolol, and untreated groups. The data is displayed as mean values with standard error (SEM). A highly significant difference (***p<0.001) was observed through one-way ANOVA and Bonferroni multiple comparison test.

Serum MDA levels were compared among the vehicle, doxorubicin, doxorubicin with nebivolol, and untreated groups. The data is displayed as mean values with standard error (SEM). A highly significant difference (***p<0.001) was detected through one-way ANOVA and Bonferroni multiple comparison test.

Cardiac TAC levels were compared among the vehicle, doxorubicin, doxorubicin with nebivolol, and untreated groups. The data is displayed as mean values with standard error (SEM). A highly significant difference (***p<0.001) was detected through one-way ANOVA and Bonferroni multiple comparison test.

Caspase 3 levels were compared among the vehicle, doxorubicin, doxorubicin with nebivolol, and untreated groups. The data is displayed as mean values with standard error (SEM). A highly significant difference (***p<0.001) was detected through one-way ANOVA and Bonferroni multiple comparison test.

Bcl-2 levels were compared among the vehicle, doxorubicin, doxorubicin with nebivolol, and untreated groups. The data is displayed as mean values with standard error (SEM). A highly significant difference (***p<0.001) was detected through one-way ANOVA and Bonferroni multiple comparison test.

## DISCUSSION

Doxorubicin is a highly efficacious cytotoxic chemotherapeutic drug with anti-tumor properties, capable of being administered either as a monotherapy or in conjunction with other therapeutic agents to eliminate cancerous cells effectively [23]. In order to mitigate the cardiotoxic effects of doxorubicin while preserving its cytotoxicity against cancer cells, novel approaches have been investigated. Nevertheless, the development of clinically effective preventive medication remains elusive [11]. The development and progression of several cardiac disorders have been associated with the presence of inflammatory cytokines, including IL-1β and TNF-α. These cytokines have been found to induce negative inotropic effects and have detrimental impacts on left ventricular remodeling [24]. Doxorubicin induces a series of inflammatory processes within the myocardium by activating NF-B and upregulating various pro-inflammatory cytokines, including TNF-α [25]. The etiology of doxorubicin-induced cardiomyopathy has been identified as a progressive increase in pro-inflammatory cytokines inside the heart tissue [26]. Similarly, the findings of the present investigation support the notion of an inflammatory mechanism underlying doxorubicin-induced cardiotoxicity, as seen by a notable elevation in cardiac inflammatory biomarkers in the doxorubicin-treated group relative to the control group. These findings exhibit similarities with the results of earlier studies. In the present investigation, it was observed that the administration of nebivolol resulted in a notable reduction in the upregulation of pro-inflammatory markers, namely IL-1β and TNF-α, in rats. This effect was particularly evident when comparing the nebivolol-treated group to the doxorubicin-induced group without treatment. This shows that nebivolol protects against the cardiotoxicity caused by doxorubicin. Doxorubicin induces substantial myocyte destruction, according to several studies. Toxic consequences of doxorubicin include multifocal patchy regions, myofibrillar loss, and vacuolated myocyte. In the majority of instances, fibrotic foci predominate, with areas of acute injury being rare, if present at all. It is possible to discover necrotic cardiomyocytes foci [20]. The study conducted by Wanas ElShabrawy demonstrated that the administration of nebivolol in a rat model of nephrotoxicity resulted in a significant reduction in plasma levels of TNF-α and IL-1β when compared to a group of rats induced with nephrotoxicity but left untreated with cyclophosphamide [27]. A separate animal investigation revealed that nebivolol exhibits a cardioprotective impact, while doxorubicin-induced cardiotoxicity leads to increased levels of pro-inflammatory cytokines, specifically TNF-α and IL-1β [28]. Our findings contradict those of Górska *et al*., who claimed that nebivolol had no effect on serum TNF-α levels in hypertensive rats [29]. Furthermore, Chiosi *et al*. found that nebivolol medication for heart failure patients did not affect serum cytokine concentrations, including TNF-α [30]. The differences could have resulted from sampling the myocardial rather than the plasma. Earlier studies have demonstrated that nebivolol has antioxidant activity [31-33]. The role of oxidative stress in doxorubicin-induced cardiotoxicity has been well-documented and proven. As a result, one of the objectives of this study was to evaluate the antioxidant capacity of nebivolol in doxorubicin-treated rats. The research findings revealed that the administration of nebivolol resulted in a notable decrease in lipid peroxidation and the preservation of antioxidant capacity in cardiac tissue. This was evidenced by the lower levels of MDA and the higher levels of TAC observed in rats treated with nebivolol and doxorubicin compared to rats treated with doxorubicin alone. Nebivolol, a pharmacological agent with selective β-blocking properties and the ability to donate nitric oxide, has been shown to improve cardiac damage induced by antineoplastic agents [34]. Moreover, a recent study showed that nebivolol reduced MDA levels and increased TAC levels in male rats with hepatotoxicity [35]. The neuroprotective properties of nebivolol were further evidenced by its application in safeguarding the brain from ischemia/reperfusion (I/R) injury in a rat model of cerebral ischemia [36]. Similarly, in another study, nebivolol protected the kidneys of rats from damage caused by thermal ischemia/reperfusion, and it caused a significant drop in kidney MDA levels [37]. A recent study revealed that the combination of nebivolol and chrysin demonstrated a potentially beneficial protective effect against liver injury generated by ischemia/reperfusion (I/R). This effect is believed to be attributed to reducing oxidative stress and elevating nitric oxide levels [38]. The present study showed that the group treated with nebivolol + DOX exhibited a decrease in caspase-3 levels and an increase in Bcl-2 levels in cardiac tissue, as compared to the group treated with DOX. This finding aligns with previous research conducted by Labib and colleagues [28], which demonstrated that nebivolol significantly reduces caspases 3 and increases Bcl-2 in a rat model of ulcerative colitis compared to an untreated induced group [39]. Mercanoglu *et al*. revealed that nebivolol suppresses myocardial apoptosis in rats by reducing reactive oxygen species formation, hence limiting infarct zone extension and aiding in maintaining left ventricular function after myocardial infarction [40]. In rats with doxorubicin-induced cardiotoxicity, nebivolol decreased myocardial alterations. This could pave the way for promising new approaches to preventing and treating anthracycline-induced cardiotoxicity in chemotherapy patients, such as those with breast cancer. Consistent findings were documented previously. Mohamed and Kassem, for example, reported that treated rats exhibited considerably fewer histological changes in their cardiac tissues than doxorubicin-treated animals, indicating that nebivolol had a cardio-protective effect [28]. Furthermore, a significant decrease in the severity of the muscular injury was seen, accompanied by substantial cytoplasm vacuolization and a small presence of inflammation. [28]. The inhibitory effects of nebivolol on apoptosis were observed in the cardiac tissue, specifically in the final stage. This was evidenced by a decrease in the release caspase-3 and an increase in Bcl-2 levels. Additionally, nebivolol was found to affect the oxidative pathway, as indicated by a reduction in lipid peroxidation and the preservation of cardiac antioxidant status. Nebivolol exhibited inhibitory effects on the inflammatory response inside the myocardium, as evidenced by reduced levels of TNF-α and IL-1β. These findings may provide a mechanistic rationale for the cardioprotective effect of nebivolol in doxorubicin-induced cardiomyopathy.

## CONCLUSION

In summary, it can be inferred that nebivolol exhibited a significant reduction in the cardiotoxic effects induced by doxorubicin in rats. This effect was likely attributed to its influence on several markers such as TNF-α, IL-1β, MDA, TAC, Bcl-2, and caspase-3. Further investigation is recommended to ascertain the potential of nebivolol in conferring protection against further anticancer drugs and explore the possibility of other beta-blockers serving as protective agents.
